# Metformin-associated Severe Lactic Acidosis in the Setting of Acute Kidney Injury

**DOI:** 10.7759/cureus.3897

**Published:** 2019-01-16

**Authors:** Hassaan Iftikhar, Maryam Saleem, Anand Kaji

**Affiliations:** 1 Internal Medicine, Seton Hall University-St. Francis Medical Center, Trenton, USA; 2 Internal Medicine, Waterbury Hospital, Waterbury, USA

**Keywords:** metformin, lactic acidosis, acute kidney injury, diabetes mellitus

## Abstract

Metformin is a first-line biguanide agent for the treatment of diabetes mellitus. It has been known to be associated with lactic acidosis. This side effect especially worsens when being used with other medications affecting the kidney such as angiotensin-converting enzyme inhibitors (ACEi) and loop diuretics. This is a case of a 63-year-old male who suffered from metformin-induced lactic acidosis and underwent hemodialysis for treatment.

## Introduction

Metformin is a biguanide medication used as first-line therapy for the treatment of diabetes mellitus type II. The well-known adverse effect of metformin is lactic acidosis, but it is rarely reported in the literature review of case reports. The assumption is that the lactic acidosis occurs from a failure of the kidneys to clear the medication from the body. We report the case of a patient who was admitted for profound lethargy and altered mental status and was found to have an elevated lactic acidosis level secondary to metformin.

## Case presentation

A 63-year-old male, with a past medical history of type II diabetes mellitus, hypertension, chronic kidney disease stage G3a, and heart failure with preserved ejection fraction, presented with generalized lethargy and weakness. His history, obtained from his family, revealed that he was taking his home medications: metformin 1000 mg twice daily, lisinopril 10 mg once daily, and furosemide 40 mg once daily but not eating or drinking adequately at home due to a lack of money. On physical examination, he was found to be confused and in a state of lethargy. He had a dry oral mucosa and scored 14/15 on the Glasgow Coma Scale. The vitals were a blood pressure of 70/40 mmHg, pulse of 70 beats/minute, a temperature of 92 degrees Fahrenheit, and a respiratory rate of 14/minute. Tables 1-2 list the major laboratory and hematology findings.

**Table 1:** Major laboratory findings BUN: blood urea nitrogen

Test	Result
Basal Metabolic Panel	
1. Sodium	147 mmol/L
2. Chloride	85 mmol/L
3. Potassium	3.1 mmol/L
4. Bicarbonate	4 mmol/L
5. BUN	97 mg/dl
6. Creatinine (Cr)	11.41 mg/dl (Baseline 1.2-1.4mg/dl)
7. Anion Gap	58
8. Albumin	4.0 g/dL
9. Lactate	18.5 mmol/L
Arterial Blood Gas	
pH	6.8
pCO_2_	20.9 mmHg
pO_2_	95.6 mmHg

**Table 2:** Hematology

Hematology	
Hemoglobin	11.7 g/dL
White blood cells	24,300/uL

His urine toxicology screen was negative and blood alcohol level was undetectable. The computed tomography (CT) scan of the head and the posterior-anterior (PA) / lateral chest X-ray were unremarkable.

The patient was admitted to the intensive care unit for a hypovolemic shock, with the resultant acute chronic renal failure secondary to hypotension worsened by the ongoing use of furosemide and lisinopril. He was treated initially with intravenous fluids and norepinephrine for blood pressure support. It was postulated that the elevated lactic acid level and anion gap level were due to hypoperfusion, likely exacerbated by the concomitant use of metformin in the setting of acute kidney injury. His altered mentation was likely due to metabolic encephalopathy secondary to high lactate.

The patient received broad-spectrum intravenous antibiotics on the day of admission, but they were discontinued later, as no source of infection was found and sepsis was ruled out. The patient also received a bicarbonate drip until the bicarbonate improved to 22 mmol/L. He subsequently underwent emergent hemodialysis. Post hemodialysis, lactic acid trended down to 8.3 mmol/L and pH improved to 7.24 with an anion gap of 35 with the first session; subsequently, lactate became undetectable, as he underwent further sessions of hemodialysis during the hospital course. His creatinine at the time of discharge was 3.12 mg/dl, bicarbonate was 31 mmol/L, the anion gap had closed, and pH was 7.34. His urine output normalized and mentation improved to GCS 15/15. He was discharged in a stable condition. His metformin, lisinopril, and furosemide were stopped, and he was advised to keep himself adequately hydrated. Outpatient creatinine was 1.4 mg/dL.

## Discussion

Metformin is a biguanide medication used as a first-line agent for new-onset diabetes mellitus patients. The well-known side effects commonly found in patients include gastrointestinal side effects, such as nausea, vomiting, and diarrhea. Metformin-induced lactic acidosis is a well-known but rare side effect with an estimated incidence of 4.3 cases per 100,000 person-years in metformin users [1]. This is especially true in patients with renal failure due to the decreased clearance of metformin from the body. It is excreted in an unmetabolized state from the proximal tubular cells of the kidneys. The plasma protein binding of metformin is minimal, so it can be hemodialyzed [2]. Metformin inhibits gluconeogenesis and increases glucose uptake by muscle and adipose tissue. It promotes the conversion of glucose to lactate and decreases hepatic gluconeogenesis from lactate, pyruvate, and alanine [3]. Metformin is commonly used in combination with angiotensin-converting enzyme inhibitors due to the prevalence of hypertension in diabetic patients and with diuretics in patients with heart failure.

In the setting of dehydration with resultant acute kidney injury, metformin can accumulate, leading to type B lactic acidosis, especially in the presence of other nephrotoxic agents (ACEi and loop diuretics) [4]. The contraindications to metformin are listed in Table 3 [5].

**Table 3:** Contraindications to metformin

Stop	If Serum Creatinine >150 umol/L
Stop	During periods of tissue hypoxia
Withdraw	For 3 days after iodine contrast medium given
Withdraw	2 days before general anesthesia is given, restart when renal function stable

The typical patient with this clinical picture will usually present with nausea, lethargy, and abdominal pain, and progress to altered mental status [6]. Serum metformin concentration is usually not available in most hospitals in the United States and doesn't correlate with disease severity appropriately in a clinical setting [7]. The use of a bicarbonate drip is still controversial and is usually limited to a pH of <7.10. However, hemodialysis with bicarbonate buffer is the treatment of choice for such patients [8]. Patients are usually treated until lactate falls below 3mmol/L and pH increases to 7.35 [9]. Also, the prognosis and outcomes of metformin-induced lactic acidosis are better than those for lactic acidosis of other etiologies as per results from a retrospective analysis [10].

## Conclusions

Our case of metformin-induced lactic acidosis shows the importance of the appropriate dosing of metformin in chronic kidney disease patients and further indicates that metformin toxicity could be worsened by ongoing dehydration in the setting of using diuretics and ACEi. It's worthwhile to review what the evidence-based practice should be and use this patient as an example of the population that actually needs dosing adjustments.

## References

[1] Salpeter SR, Greyber E, Pasternak GA, Salpeter EE (2010). Risk of fatal and nonfatal lactic acidosis with metformin use in type 2 diabetes mellitus. Cochrane Database Syst Rev.

[2] Chang CT, Chen YC, Fang JT, Huang CC (2001). Metformin-associated lactic acidosis: case reports and literature review. J Nephrol.

[3] Bailey CJ, Turner RC (1996). Metformin. N Engl J Med.

[4] Fitzgerald E, Mathieu S, Ball A (2009). Metformin associated lactic acidosis. BMJ.

[5] Jones GC, Macklin JP, Alexander WD (2003). Contraindications to the use of metformin. BMJ.

[6] Gan SC, Barr J, Arieff AI, Pearl RG (1992). Biguanide-associated lactic acidosis. Case report and review of the literature. Arch Intern Med.

[7] Vecchio S, Giampreti A, Petrolini VM (2014). Metformin accumulation: lactic acidosis and high plasmatic metformin levels in a retrospective case series of 66 patients on chronic therapy. Clin Toxicol (Phila).

[8] Heaney D, Majid A, Junor B (1997). Bicarbonate haemodialysis as a treatment of metformin overdose. Nephrol Dial Transplant.

[9] Calello DP, Liu KD, Wiegand TJ (2015). Extracorporeal treatment for metformin poisoning: systematic review and recommendations from the Extracorporeal Treatments in Poisoning Workgroup. Crit Care Med.

[10] Friesecke S, Abel P, Roser M, Felix SB, Runge S (2010). Outcome of severe lactic acidosis associated with metformin accumulation. Crit Care.

